# Coordinating Leader-Follower Supply Chain with Sustainable Green Technology Innovation on Their Fairness Concerns

**DOI:** 10.3390/ijerph14111357

**Published:** 2017-11-08

**Authors:** Bisheng Du, Qing Liu, Guiping Li

**Affiliations:** 1 School of Business, Ningbo University, Ningbo 315211, China; liuqingelse@163.com; 2 Center for Collaborative Innovation on Port Trading Cooperation and Development (Collaborative Innovation Center of Port Economics), Ningbo University, Ningbo 315211, China

**Keywords:** sustainable green technology innovation, supply chain, fairness concern, game theory

## Abstract

Sustainable green technology innovation is essential in all the stages of the supply chain development. The members of the supply chain in each stage need to invest in sustainable green technology innovation research and development. However, whether the sustainable green technology innovation investments and profits for all the members are fairness concerned is a critical factor to motivate the supply chain members. Motivated by a real business investigation, in this study, a supply chain model with one supplier and one manufacturer is analyzed. We consider fairness concerns for the supplier and the manufacturer with sustainable green technology innovation development. We derive the optimal results in both with and without fairness concern. The results indicate that fairness concerns can promote and coordinate the supply chain members without advantage inequity averseness, to invest more on their sustainable green technology innovation development.

## 1. Introduction

In the sustainable green technology innovation development, companies have to use the up-to-date methodologies from a technological, economic and social environmental viewpoint to the company’s R&D [1]. The aim of sustainable green technology innovation is to produce quality and innovative products able to reduce environmental impacts. As the current environmental challenges are on the up trend, the importance of sustainable green technology innovation is widely recognized. For example, an Eco-Innovation project “textile4textile” was launched in 2008 from the sustainability initiative by the European Commission, as a part of its Entrepreneurship and Innovation Program (EIP), to support sustainable green technology innovation among SMEs [2]. Nowadays, many companies already acknowledged that the use of sustainable green technology innovation in their development strategies and product manufacturing processes is beneficial to their business operation performances and social influences. Innumerable companies, operating in many different industries that already provide valuable out-of-the-box thinking for their sustainable products with innovations from their inventors along with their supply chain members. For instance, just to mention one of the uncountable examples, Vegan shoes are made of light but durable materials like Tyvek, which is a DuPont synthetic material made from pressed polyethylene [3].

A sustainability driven innovation can definitely offer supply chain members significant potential benefits from the upstream to the downstream [4]. However, the sustainable green technology innovation development depends not only on the supply chain members’ efforts during the whole process, but also on the fairness concerns among the supply chain members themselves. The equity and fairness, with respect to the environment and the sustainable green technology innovation development, represent, for all the members of a supply chain great challenges, such as the imbalanced supply and demand for resources, environmental disruption, workers assignments.

Motivated by the above practices, we present a study on the supply chain made-up by one supplier and one manufacturer, and characterized by both sustainable green technology innovation effort investment and fairness concerns mechanisms. We aim to find out the conditions of the optimal decisions with sustainable green technology innovation efforts provided by the supplier and the manufacturer, as well as their pricing policies for selling sustainable innovation products to their downstream customers respectively. Toward this goal, we consider the leader-follower Stackelberg game between the supplier and the manufacturer who aim to maximize their profits and utilities, first, without fairness concerns and, second, with fairness concerns. To the best of our knowledge, this paper is one of the first study streams investigating the impacts of sustainable green technology innovation efforts on the performance of the supplier and the manufacturer, and it compares the analytical results of the supplier and the manufacturer with respect to their fairness concerns. We aim to find out serval interesting managerial insights, which can reveal the value of sustainable green technology innovations and corresponding fairness concerns in a Stackelberg game supply chain. First, we find that companies have incentives to pay for the sustainable green technology innovation if they can get higher benefits from their sustainable green technology innovation efforts. Second, we examine how fairness concerns affect the performance of the supplier and the manufacturer with sustainable green technology innovation efforts. Then, third, we compare the optimal decisions of the supplier and the manufacturer with respect to their corresponding fairness concerns in the supply chain. All these findings provide important implications for the research community on the operations management with sustainable green technology innovation and research development under different channel power.

The reminder of this paper is organized as follows. In Section 2 we review the relevant research streams of analytical models with respect to sustainable green technology innovation and fairness concerns. In Section 3 we develop the mathematical model of sustainable green technology innovation without fairness concerns and conduct the analyses. In Section 4 we present the analyses for sustainable green technology innovation with fairness concerns under two scenarios (sustainable green technology innovation effort provided by the supplier and by the manufacturer). In Section 5 we present the numerical analysis and managerial implications. Concluding remarks and further research directions are given in Section 6. All the proofs are regulated to the Appendixes Appendix A and Appendix B.

## 2. Literature Review

Our study is closely related to the following two research streams: sustainable green technology innovation supply chain and fairness concerns in supply chain.

Regarding the first stream, sustainable green technology innovation supply chain, serval foregoing academic studies need to be mentioned. Aminoff and Kettunen [5] study the sustainable supply chain management (SSCM) from a circular economy perspective. They summarize the state of the art SSCM literature and list the findings from empirical data. Tunn and Dekoninck [6] study how sustainability impacts innovation in industry, help or hinder. The sustainability helps innovation with the following ways: improving packaging solutions, reducing waste—Eco-efficiency, finding more sustainable solutions for standard products, upcycling. The sustainability hinders innovation with the following ways: issues with supplies of sustainable materials, challenges of sustainable materials from a manufacturing perspective, issues with sustainable materials from a customer perspective, the cost factor, a matter of convenience for customers. Li and Shen [7] review the importance of sustainable design with the comparison of non-profit manufacturer and for-profit manufacturer. Barbosa-Póvoa [8] reviews the state-of-the-art relevant research optimizing decision making models on sustainable supply chain, and extends the classic supply chain model in a theme that guarantees costumers satisfaction and contributes positively to society and the planet. Roos [9] investigates the sustainable innovation values on the future circular material chains. Behnam and Cagliano [10] study the sustainability and innovation performances from an empirical analysis aspect. Foxon and Pearson [11] study the sustainable innovation policy of UK by implementing a case study of low carbon energy innovation and stimulating the development of the corresponding policy regime. Zijm et al. [12] collect the research work of logistics innovation and sustainability from the fields of transportation, cold store, sustainable fuels, among others. Pagell and Wu [13] provide an overview on the business implications of sustainability practices in supply chains. Wang [14] explores the sustainable green technology development of China in a global context. Boons et al. [15] organize a special issue with selected papers presented at the ERSCP-EMSU 2010 Conference. In addition, Boons and Lüdeke-Freund [16] focus on how business models and sustainable innovations interrelate, and the links between sustainable innovations and the business model concept. Cortes [17] analyzes the sustainable supply chain performance with triple bottom approach and data envelopment analysis. De Medeiros et al. [18] study the success factors of environmentally sustainable product innovation from a systematical view. Bitzer [19] and Roos [9] also examine the sustainable innovation from social responsibility and future influences.

Furthermore, also on the second stream, serval research works in fairness concerns of supply chain have to be introduced. Fairness decision has been recognized as one of the most important incentive factors that affect the executive abilities of all the related members. Therefore, it’s critical for all the decision-makers in a supply chain to learn how to confront these problems head-on and resolve them efficiently. Undoubtedly, supply chain considering fairness concerns is a hot research topic in recent years [20,21,22,23]. Fehr et al. [24] study the fairness concerns problem with respect to their competition and their collaboration from economical analysis. Later on, there are serval scholars investigate the fairness concerns in supply chain from different aspects and theoretical methods. For instance, Caliskan-Demirag et al. [25] investigates the channel coordination with nonlinear demand setting, specifically, they assume an exponential demand model and build the utility function with monetary profit and disutility with unfairness. Du et al. [26] propose a dyadic supply chain that both the supplier and the retailer have the preference of status-seeking with fairness concerns, then the optimal decisions of status-seeking behaviors are conducted under newsvendor model environment. Li and Li [27] analyze the impacts of value-added service provided by the retailer and the impacts of the retailer’s fairness concerns on pricing strategies. Liu et al. [28] extends the study to model the order allocation with fairness concerns and to assume the demand information can be updated. Chen, Zhou and Zhong [29] investigate the effect of fairness concerns on financial supply chain with buyback guarantee and conduct the optimal solutions of pricing and ordering. Kim, Lee and Lee [30] investigate the innovation performance with the buyer’s power and fairness concerns. Specifically, they assume that the fairness and power have impacts on innovation performance from the social capital accumulation’s perspective. Li et al. [31] establish analytical models with fairness concerns to study the supplier encroachment phenomenon while the wholesale suppliers open direct channels to compete with their downstream retailers in the e-commerce era. Pu, Gong and Han [32] build a feasible incentive contract to analyze the manufacturer and the corresponding fairness-sensitive retailers. They develop a profit-sharing contract to coordinate supply chain members with respect to their marketing efforts. In addition, the research of supply chain with fairness concerns extend to several interesting subfields like quantity discount contracts [33], asymmetrical cost information [34] and retail services [27].

## 3. Problem Formulation

### 3.1. Assumption and Notation

We consider a supply chain system with one supplier (hereafter he refers to the supplier) and one manufacturer (hereafter she refers to the manufacturer). The supplier sells his products to the manufacturer with a certain wholesale price, while the manufacturer sells her products to the market with a certain selling price. In this system, both the supplier and the manufacturer should commit themselves to contribute to their sustainable green technology innovation development. A sustainable innovative product with sustainable innovative functions attract more and different customers each of them with its own preferences. We assume that the demand function is sensitive to the selling price and the sustainable green technology innovation efforts provided by the supplier and the manufacturer. We consider the following market demand function

(1) $$D\left(p,i_{s},i_{m}\right)=A-Bp+k_{s}i_{s}+k_{m}i_{m}$$

where *A* is a constant parameter of the market base which is no affected by either price or sustainable green technology innovation efforts, *B* is the sensitive coefficient to the selling price. *A*, *B* are positive, and A>Bp, which means that this is a rational market, and the general market base is controlled under a rational pricing theme. $k_{s}$ is the coefficient parameter of sustainable green technology innovation effort provided by the supplier. $k_{m}$ is the coefficient parameter of sustainable green technology innovation effort provided by the manufacturer. *p* is the unit selling price determining by the manufacturer, $i_{s}$ is the sustainable green technology innovation effort provided by the supplier, $i_{m}$ is the sustainable green technology innovation effort provided by the manufacturer.

When $B\geq k_{s}$, it means that the market is more sensitive to the selling price than the sustainable green technology innovation effort provided by the supplier; analogously, when $B\geq k_{m}$, the market is more sensitive to the selling price than the sustainable green technology innovation effort provided by the manufacturer. When $B<k_{s}$ (or $B<k_{m}$), we get the opposite of the above.

The market demand function has the following special features:

- When there is no sustainable green technology innovation effort provided by the manufacturer and only the supplier plunges sustainable green technology innovation effort into the firm’s R&D activities, then the market demand function is $D\left(p,i_{s}\right)=A-Bp+k_{s}i_{s}$.
- When there is no sustainable green technology innovation effort provided by the supplier and only the manufacturer plunges sustainable green technology innovation effort into the firm’s R&D activities, then the market demand function is $D\left(p,i_{m}\right)=A-Bp+k_{m}i_{m}$.

The investment function for sustainable green technology innovation effort provided by the supplier, $C(i_{s})$ is strictly increasing in $i_{s}$, and the marginal cost of the sustainable green technology innovation effort provided by the supplier is strictly increasing in $i_{s}$, i.e., $\partial C\left(i_{s}\right)/\partial i_{s}>0$ and $\partial^{2}C\left(i_{s}\right)/\partial i_{s}^{2}>0$. In order to obtain more closed-form analytical insights, we assume that the cost of investment for sustainable green technology innovation effort provided by the supplier is a quadratic function, i.e., $C\left(i_{s}\right)=H_{s}\cdot i_{s}^{2}$, where $H_{s}$ is a scaling parameter and refers to the investment sensitivity of sustainable green technology innovation efforts provided by the supplier. Notice that this form of quadratic innovative effort cost function has been widely used in the operations management literature, for instance, [35]. Analogously, the investment function for sustainable green technology innovation effort provided by the manufacturer, $C(i_{m})$ is strictly increasing in $i_{m}$, and the marginal cost of the sustainable green technology innovation effort provided by the manufacturer is strictly increasing in $i_{m}$, i.e., $\partial C\left(i_{m}\right)/\partial i_{m}>0$ and $\partial^{2}C\left(i_{m}\right)/\partial i_{m}^{2}>0$. We assume $C\left(i_{m}\right)=H_{m}\cdot i_{m}^{2}$, where $H_{m}$ is a scaling parameter refers to investment sensitivity of sustainable green technology innovation efforts provided by the manufacturer.

The following assumptions are needed in our model.

*A1:*  All members of the supply chain are rational and pursue profit maximization and utility maximization.
*A2:*  The supplier and the manufacturer of the supply chain are running under symmetric information in their sustainable green technology innovation efforts.
*A3:*  p>w≥c, where the unit retailing price *p* is greater than the unit wholesale price *w*, and the unit wholesale price *w* is no less than the unit cost *c*.
*A4:*  Without the fairness concerns, when the sustainable green technology innovation efforts provided by the supplier, the manufacturer and both of them, the following assumptions hold.
  - $8BH_{s}>k_{s}^{2}$.
  - $4BH_{m}>k_{m}^{2}$, or we assume $\Delta_{m}=4BH_{m}-k_{m}^{2}>0$.
  - $2H_{s}\left(4BH_{m}-k_{m}^{2}\right)-H_{m}k_{s}^{2}>0$.
*A5:*  With the fairness concerns, when the sustainable green technology innovation efforts provided by the supplier, the manufacturer and both of them, the following assumptions hold.
  - $\left(2-3\theta \right)H_{s}\Delta_{m}-\left(1-\theta \right)H_{m}k_{s}^{2}>0$.
  - $2\left(1+2\lambda \right)\left(BH_{m}-k_{m}^{2}\right)H_{s}\Delta_{m}+\left(1+\lambda \right)BH_{m}^{2}k_{s}^{2}>0$.

### 3.2. Benchmarking Sustainable Green Technology Innovation Effort Model without Fairness Concerns

#### 3.2.1. SGTIE Only by the Supplier

In this scenario, sustainable green technology innovation effort (SGTIE) is only provided by the supplier. Therefore, the supplier decides his wholesale price *w* and his sustainable green technology innovation effort $i_{s}$ while the manufacturer decides only her selling price *p*. Then the supplier’s profit function can be formulated as follows,

(2) $$\begin{array}{cc} \max_{w,i_{s}}\Pi_{s}^{N1} & =\left(w-c\right)D\left(p,i_{s}\right)-H_{s}\cdot i_{s}^{2}\\ & =\left(w-c\right)\left(A-Bp+k_{s}i_{s}\right)-H_{s}\cdot i_{s}^{2} \end{array}$$

The superscript N1 represents the first case without fairness concerns. The supplier’s profit includes two parts. The first part is the supplier’s gross profit from selling the product to the manufacturer. The second part is the cost of investment for sustainable green technology innovation effort provided by the supplier.

The manufacturer’s profit function can be formulated as follows,

(3) $$\begin{array}{cc} \max_{p}\Pi_{m}^{N1} & =\left(p-w\right)D\left(p,i_{s}\right)\\ & =\left(p-w\right)\left(A-Bp+k_{s}i_{s}\right) \end{array}$$

The manufacturer’s profit includes only one part. That is the manufacturer’s gross profit from selling the product to the customers in the market.

#### 3.2.2. SGTIE Only by the Manufacturer

In this scenario, sustainable green technology innovation effort (SGTIE) is only provided by the manufacturer. Therefore, the supplier only decides his wholesale price *w* while the manufacturer decides her selling price *p* and her sustainable green technology innovation effort $i_{m}$. Then the supplier’s profit function can be formulated as follows,

(4) $$\begin{array}{cc} \max_{w}\Pi_{s}^{N2} & =\left(w-c\right)D\left(p,i_{m}\right)\\ & =\left(w-c\right)\left(A-Bp+k_{m}i_{m}\right) \end{array}$$

The superscript N2 represents the second case without fairness concerns. The supplier’s profit includes only one part. That is the supplier’s gross profit from selling the product to the manufacturer.

The manufacturer’s profit function can be formulated as follows,

(5) $$\begin{array}{cc} \max_{p,i_{m}}\Pi_{m}^{N2} & =\left(p-w\right)D\left(p,i_{m}\right)-H_{m}\cdot i_{m}^{2}\\ & =\left(p-w\right)\left(A-Bp+k_{m}i_{m}\right)-H_{m}\cdot i_{m}^{2} \end{array}$$

The manufacturer’s profit includes two parts. The first part is the manufacturer’s gross profit from selling the product to the customers in the market. The second part is the cost of investment for sustainable green technology innovation effort provided by the manufacturer.

#### 3.2.3. SGTIE by Both the Supplier and the Manufacturer

In this scenario, sustainable green technology innovation efforts are provided by both the supplier and the manufacturer. Therefore, the supplier decides his wholesale price *w* and his sustainable green technology innovation effort $i_{s}$, and the manufacturer decides her selling price *p* and her sustainable green technology innovation effort $i_{m}$. Both the supplier and the manufacturer are rational players, their rational behaviors reflect on their utilities. Therefore, their utilities are exact equal to their profits respectively. Then the supplier’s profit (utility) function in its general form can be formulated as follows,

(6) $$\begin{array}{cc} \max_{w,i_{s}}\Pi_{s}^{N3} & =\left(w-c\right)D\left(p,i_{s},i_{m}\right)-H_{s}\cdot i_{s}^{2}\\ & =\left(w-c\right)\left(A-Bp+k_{s}i_{s}+k_{m}i_{m}\right)-H_{s}\cdot i_{s}^{2} \end{array}$$

The superscript N3 represents the third case without fairness concerns. The supplier’s profit (utility) includes two parts. The first part is the supplier’s gross profit from selling the product to the manufacturer. The second part is the cost of investment for sustainable green technology innovation effort provided by the supplier.

The manufacturer’s profit (utility) function in its general form can be formulated as follows,

(7) $$\begin{array}{cc} \max_{p,i_{m}}\Pi_{m}^{N3} & =\left(p-w\right)D\left(p,i_{s},i_{m}\right)-H_{m}\cdot i_{m}^{2}\\ & =\left(p-w\right)\left(A-Bp+k_{s}i_{s}+k_{m}i_{m}\right)-H_{m}\cdot i_{m}^{2} \end{array}$$

The manufacturer’s profit (utility) includes two parts. The first part is the manufacturer’s gross profit from selling the product to the customers in the market. The second part is the cost of investment for sustainable green technology innovation effort provided by the manufacturer.

We conduct the analytical analysis assuming that the supplier with sustainable green technology innovation effort is the leader, while the manufacturer with sustainable green technology innovation effort is the follower. We examine the optimal pricing decisions and the optimal sustainable green technology innovation effort decisions for the supplier and the manufacturer. First, we derive the Stackelberg game with the sustainable green technology innovation effort only provided by the supplier. Second, we derive the Stackelberg game with the sustainable green technology innovation effort only provided by the manufacturer. Finally, we derive the Stackelberg game with the sustainable green technology innovation efforts provided by both of them.

#### 3.2.4. SGTIE Effects on the Members

Comparing the three scenarios above with the sustainable green technology innovation efforts conducted from different entities, we set the following theorems.

**Theorem** **1.** *In the scenario of sustainable green technology innovation effort only provided by the supplier, the manufacturer’s optimal decision is $p^{N1*}=\frac{6AH_{s}+2BcH_{s}-ck_{s}^{2}}{8BH_{s}-k_{s}^{2}}$, the supplier’s optimal decisions are $w^{N1*}=\frac{4H_{s}\left(A+Bc\right)-ck_{s}^{2}}{8BH_{s}-k_{s}^{2}}$ and $i_{s}^{N1*}=\frac{\left(A-Bc\right)k_{s}}{8BH_{s}-k_{s}^{2}}$. The optimal profit of the supplier is $\Pi_{s}^{N1*}=H_{s}\frac{{\left(A-Bc\right)}^{2}}{8BH_{s}-k_{s}^{2}}$ and the optimal profit of the manufacturer is $\Pi_{m}^{N1*}=4BH_{s}^{2}\frac{{\left(A-Bc\right)}^{2}}{{\left(8BH_{s}-k_{s}^{2}\right)}^{2}}$*.

**Theorem** **2.** *In the scenario of sustainable green technology innovation effort only provided by the manufacturer, the manufacturer’s optimal decision are $p^{N2*}=\frac{2BH_{m}\left(3A+Bc\right)-k_{m}^{2}\left(A+Bc\right)}{2B(4BH_{m}-k_{m}^{2})}$ and $i_{m}^{N2*}=\frac{k_{m}}{2}\frac{A-Bc}{4BH_{m}-k_{m}^{2}}$, the supplier’s optimal decision is $w^{N2*}=\frac{A+Bc}{2B}$. The optimal profit of the supplier is $\Pi_{s}^{N2*}=\frac{H_{m}}{2}\frac{{\left(A-Bc\right)}^{2}}{4BH_{m}-k_{m}^{2}}$ and the optimal profit of the manufacturer is $\Pi_{m}^{N2*}=\frac{H_{m}}{4}\frac{{\left(A-Bc\right)}^{2}}{4BH_{m}-k_{m}^{2}}$*.

**Theorem** **3.** *In the scenario of sustainable green technology innovation effort provided by both the supplier and the manufacturer, which means that both of them are rational players in the supply chain. Their utilities equal to their profits. Therefore, the manufacturer’s optimal decisions of selling price and sustainable green technology innovation effort are $p^{N3*}=\frac{1}{B}\frac{AH_{s}\left(6BH_{m}-k_{m}^{2}\right)+Bc\left(2BH_{s}H_{m}-H_{s}k_{m}^{2}-H_{m}k_{s}^{2}\right)}{2H_{s}\left(4BH_{m}-k_{m}^{2}\right)-H_{m}k_{s}^{2}}$ and $i_{m}^{N3*}=\frac{H_{s}k_{m}\left(A-Bc\right)}{2H_{s}\left(4BH_{m}-k_{m}^{2}\right)-H_{m}k_{s}^{2}}$, the supplier’s optimal decisions of wholesale price and sustainable green technology innovation effort are $w^{N3*}=\frac{1}{B}\frac{H_{s}\left(A+Bc\right)\left(4BH_{m}-k_{m}^{2}\right)-BcH_{m}k_{s}^{2}}{2H_{s}\left(4BH_{m}-k_{m}^{2}\right)-H_{m}k_{s}^{2}}$ and $i_{s}^{N3*}=\frac{H_{m}k_{s}\left(A-Bc\right)}{2H_{s}\left(4BH_{m}-k_{m}^{2}\right)-H_{m}k_{s}^{2}}$. The optimal profit (utility) of the supplier is $\Pi_{s}^{N3*}=\frac{H_{m}}{2}\frac{{\left(A-Bc\right)}^{2}}{4BH_{m}-k_{m}^{2}}$ and the optimal profit (utility) of the manufacturer is $\Pi_{m}^{N3*}=\frac{H_{m}}{4}\frac{{\left(A-Bc\right)}^{2}}{4BH_{m}-k_{m}^{2}}$*.

It’s easy to get the analytical results of the supplier and the manufacturer without sustainable green technology innovation effort and without fairness concerns. The market demand function is $D_{0}\left(p\right)=A-Bp$. The supplier’s profit function is $\Pi_{s}^{0}\left(w\right)=\left(w-c\right)\left(A-Bp\right)$, while the manufacturer’s profit function is $\Pi_{m}^{0}\left(p\right)=\left(p-w\right)\left(A-Bp\right)$. The optimal decisions under Stackelberg game are $p^{0*}=\frac{3A+Bc}{4B}$, $w^{0*}=\frac{A+Bc}{2B}$. The optimal demand is $D_{0}^{*}=\frac{A-Bc}{4}$. The optimal profit values under Stackelberg game for the supplier and the manufacturer are $\Pi_{s}^{0*}=\frac{{\left(A-Bc\right)}^{2}}{8B}$, $\Pi_{m}^{0*}=\frac{{\left(A-Bc\right)}^{2}}{16B}$, respectively.

**Proposition** **1.** *The profit values of the supplier and the manufacturer with sustainable green technology innovation efforts, are definitely higher than their corresponding profit values without sustainable green technology innovation efforts. $\Pi_{s}^{N1*}>\Pi_{s}^{0*}$, $\Pi_{s}^{N2*}>\Pi_{s}^{0*}$, $\Pi_{m}^{N1*}>\Pi_{m}^{0*}$, $\Pi_{m}^{N2*}>\Pi_{m}^{0*}$*.

This proposition implies the following insights:

When the sustainable green technology innovation effort is only provided by the supplier, the manufacturer could get more profits from the externalities of the sustainable green technology innovation effort investment. On the other hand, when the sustainable green technology innovation effort is only provided by the manufacturer, the supplier could also get more profits from the externalities of the sustainable green technology innovation effort investment.

When the sustainable green technology innovation effort only provided by the supplier (or the manufacturer), there is a clear monotonic positive association between the optimal sustainable green technology innovation effort of the supplier (or the manufacturer) and the market’s base. That means, when the market’s base is increasing, then the optimal sustainable green technology innovation effort of the supplier (or the manufacturer) should increase as well.

Both the supplier and the manufacturer have incentives to join the sustainable green technology innovation development because if the supplier joins it but the manufacturer does not, the manufacturer will lose the potential customers demands, and on the other hand, if the manufacturer joins it but the supplier does not, the supplier will lose the potential customers demands.

## 4. The Decisions with Fairness Concerns

We conduct the analytical analysis by choosing the supplier’s fairness utility function with sustainable green technology innovation effort as the leader, and the manufacturer’s fairness utility function with sustainable green technology innovation effort as the follower. We examine the optimal pricing decisions and the optimal sustainable green technology innovation effort decisions for the supplier’s fairness utility function and the manufacturer’s fairness utility function. First, we derive the Stackelberg game when only the supplier has concerns about fairness. Second, we derive the Stackelberg game when only the manufacturer has concerns about fairness.

### 4.1. The Supplier Concerns about Fairness

Based on the model established by [24], the utility function of the supplier with fairness concerns can be described as follows,

(8) $$\begin{array}{c} \mu \left(\Pi_{s}\right)=\Pi_{s}-\lambda \max\left(\Pi_{m}-\Pi_{s},0\right)-\theta \max\left(\Pi_{s}-\Pi_{m},0\right) \end{array}$$

where the coefficients λ and θ representing the disadvantage and advantage inequity averseness respectively. As we expressed in the above section that the supplier with sustainable green technology innovation effort is the leader, while the manufacturer with sustainable green technology innovation effort is the follower. In addition, we compare the analytical optimal results in Table 1, we find that $\Pi_{s}^{*}>\Pi_{m}^{*}$. Therefore, the utility function of the supplier with fairness concerns can be rewritten as the follow,

(9) $$\begin{array}{c} \mu^{F1}\left(\Pi_{s}\right)=\Pi_{s}-\theta \left(\Pi_{s}-\Pi_{m}\right) \end{array}$$

in which the superscript F1 stands for the first case with fairness concerns.

The analytical model in which only the supplier is concerned about fairness can be expressed as,

(10) $$\begin{array}{cc} \max\; & \mu^{F1}\left(\Pi_{s}\right)=\Pi_{s}-\theta \left(\Pi_{s}-\Pi_{m}\right) \end{array}$$

(11) $$\begin{array}{cc} s.t. & \max\;\mu^{F1}\left(\Pi_{m}\right)=\Pi_{m} \end{array}$$

**Theorem** **4.** *In the scenario of sustainable green technology innovation effort in which only the supplier concerning about fairness, the manufacturer’s optimal decisions are*

$$p^{F1*}=\frac{1}{B}\frac{AH_{s}\left[2\left(3-5\theta \right)BH_{m}+\left(1-2\theta \right)k_{m}^{2}\right]+Bc\left(1-\theta \right)\left[2BH_{m}H_{s}-H_{s}k_{m}^{2}-H_{m}k_{s}^{2}\right]}{\left(2-3\theta \right)H_{s}\Delta_{m}-\left(1-\theta \right)H_{m}k_{s}^{2}}$$

*and*

$$i_{m}^{F1*}=\frac{\left(1-\theta \right)\left(A-Bc\right)H_{s}k_{m}}{\left(2-3\theta \right)H_{s}\Delta_{m}-\left(1-\theta \right)H_{m}k_{s}^{2}}$$

*while the supplier’s optimal decisions are*

$$w^{F1*}=\frac{1}{B}\frac{\left(1-2\theta \right)AH_{s}\Delta_{m}+\left(1-\theta \right)BcH_{s}\Delta_{m}-\left(1-\theta \right)BcH_{m}k_{s}^{2}}{\left(2-3\theta \right)H_{s}\Delta_{m}-\left(1-\theta \right)H_{m}k_{s}^{2}}$$

*and*

$$i_{s}^{F1*}=\frac{\left(1-\theta \right)H_{m}k_{s}\left(A-Bc\right)}{\left(2-3\theta \right)H_{s}\Delta_{m}-\left(1-\theta \right)H_{m}k_{s}^{2}}$$

*Then the supplier’s optimal utility and the manufacturer’s optimal utility can be obtained from the optimal decision variables above.*

**Proposition** **2.** *If there is only the supplier concerning* *about the fairness, then we have the following:*

*The manufacturer’s sustainable green technology innovation effort*

(12) $$\begin{array}{c} \frac{\partial i_{m}^{F1*}}{\partial \theta }=\frac{\left(A-Bc\right)H_{s}^{2}k_{m}\Delta_{m}}{{\left[\left(2-3\theta \right)H_{s}\Delta_{m}-\left(1-\theta \right)H_{m}k_{s}^{2}\right]}^{2}}>0 \end{array}$$

*The supplier’s sustainable green technology innovation effort*

(13) $$\begin{array}{c} \frac{\partial i_{s}^{F1*}}{\partial \theta }=\frac{\left(A-Bc\right)H_{s}H_{m}k_{s}\Delta_{m}}{{\left[\left(2-3\theta \right)H_{s}\Delta_{m}-\left(1-\theta \right)H_{m}k_{s}^{2}\right]}^{2}}>0 \end{array}$$

This proposition implies that when only the supplier is concerned about fairness, and all other parameters are kept unchanged, then the inequity attitude parameter θ given by the supplier will increase and the sustainable green technology innovation development of the manufacturer will provide more efforts. On the other hand, the inequity attitude parameter θ will also increase and the supplier provides more efforts on the sustainable green technology innovation development.

### 4.2. The Manufacturer Concerns about Fairness

Analogously, based on the model established by [24], the utility function of the manufacturer with fairness concerns can be described as follows,

(14) $$\begin{array}{c} \mu \left(\Pi_{m}\right)=\Pi_{m}-\theta \max\left(\Pi_{m}-\Pi_{s},0\right)-\lambda \max\left(\Pi_{s}-\Pi_{m},0\right) \end{array}$$

where the coefficients λ and θ representing the disadvantage and advantage inequity averseness respectively. As we expressed in the above section that the supplier with sustainable green technology innovation effort is the leader, while the manufacturer with sustainable green technology innovation effort is the follower. Furthermore, by comparing the analytical optimal results in Table 1, we find out that $\Pi_{s}>\Pi_{m}$. Therefore, the utility function of the manufacturer with fairness concerns can be rewritten as follows,

(15) $$\begin{array}{c} \mu^{F2}\left(\Pi_{m}\right)=\Pi_{m}-\lambda \left(\Pi_{s}-\Pi_{m}\right)=\left(1+\lambda \right)\Pi_{m}-\lambda \Pi_{s} \end{array}$$

in which the superscript F2 stands for the second case with fairness concerns.

The analytical model in which only the manufacturer has fairness concerns can be expressed as,

(16) $$\begin{array}{cc} \max\; & \mu^{F2}\left(\Pi_{s}\right)=\Pi_{s} \end{array}$$

(17) $$\begin{array}{cc} s.t. & \max\;\mu^{F2}\left(\Pi_{m}\right)=\left(1+\lambda \right)\Pi_{m}-\lambda \Pi_{s} \end{array}$$

**Theorem** **5.** *In the scenario of sustainable green technology innovation effort in which only the manufacturer has concerns about fairness, the manufacturer’s optimal decisions are*

$$p^{F2*}=\frac{\left\{\begin{array}{c} \left(1-\lambda \right)\left(1+2\lambda \right)\left(2BH_{m}-k_{m}^{2}\right)\left(BH_{m}-k_{m}^{2}\right)H_{s}\Delta_{m}c+{\left(1+\lambda \right)}^{2}\left(2BH_{m}-k_{m}^{2}\right)H_{m}^{2}k_{s}^{2}c\\ +5\left(1+\lambda \right)\left(1+2\lambda \right)\left(2BH_{m}-k_{m}^{2}\right)BcH_{m}^{2}k_{s}^{2}-4\left(1+\lambda \right)\left(1+2\lambda \right)ABH_{m}^{2}H_{s}\Delta_{m}\\ +4{\left(1+\lambda \right)}^{2}ABH_{m}^{3}k_{s}^{2}-2\left(1+\lambda \right)\left(1+3\lambda \right)\left(BH_{m}-k_{m}^{2}\right)BcH_{m}^{2}k_{s}^{2} \end{array}\right\}}{\left(1+\lambda \right)\Delta_{m}\left[2\left(1+2\lambda \right)\left(BH_{m}-k_{m}^{2}\right)H_{s}\Delta_{m}+\left(1+\lambda \right)BH_{m}^{2}k_{s}^{2}\right]}$$

*and*

$$i_{m}^{F2*}=k_{m}\frac{\left\{\begin{array}{c} 2\left(1+\lambda \right)\left(1+2\lambda \right)\left(BH_{m}-k_{m}^{2}\right)AH_{s}\Delta_{m}+2{\left(1+\lambda \right)}^{2}ABH_{m}^{2}k_{s}^{2}\\ -\left(1+\lambda \right)\left(1+3\lambda \right)\left(BH_{m}-k_{m}^{2}\right)BcH_{m}k_{s}^{2}-\left(1-\lambda \right)\left(1+2\lambda \right)\left(BH_{m}-k_{m}^{2}\right)BcH_{s}\Delta_{m}\\ +{\left(1+\lambda \right)}^{2}B^{2}cH_{m}^{2}k_{s}^{2}-\left(1+\lambda \right)\left(1+2\lambda \right)ABH_{m}H_{s}\Delta_{m} \end{array}\right\}}{\left(1+\lambda \right)\Delta_{m}\left[2\left(1+2\lambda \right)\left(BH_{m}-k_{m}^{2}\right)H_{s}\Delta_{m}+\left(1+\lambda \right)BH_{m}^{2}k_{s}^{2}\right]}$$

*while the supplier’s optimal decisions are*

$$w^{F2*}=\frac{\left(1+\lambda \right)\left[\left(BH_{m}-k_{m}^{2}\right)H_{s}\Delta_{m}+BH_{m}^{2}k_{s}^{2}\right]c+\left(1+\lambda \right)AH_{m}H_{s}\Delta_{m}}{2\left(1+2\lambda \right)\left(BH_{m}-k_{m}^{2}\right)H_{s}\Delta_{m}+\left(1+\lambda \right)BH_{m}^{2}k_{s}^{2}}$$

*and*

$$i_{s}^{F2*}=BH_{m}k_{s}\frac{\left(1+\lambda \right)AH_{m}-\left(1+3\lambda \right)\left(BH_{m}-k_{m}^{2}\right)c}{2\left(1+2\lambda \right)\left(BH_{m}-k_{m}^{2}\right)H_{s}\Delta_{m}+\left(1+\lambda \right)BH_{m}^{2}k_{s}^{2}}$$

*Then the supplier’s optimal utility and the manufacturer’s optimal utility can be obtained from the optimal decision variables above*.

**Proposition** **3.** *If there is only the manufacturer concerning* *about the fairness, then we have the following:*

*The manufacturer’s sustainable green technology innovation effort*

$$\begin{array}{c} \frac{\partial i_{m}^{F2*}}{\partial \lambda }=\frac{\left(A-Bc\right)H_{s}H_{m}k_{s}\Delta_{m}}{{\left[\left(2-3\theta \right)H_{s}\Delta_{m}-\left(1-\theta \right)H_{m}k_{s}^{2}\right]}^{2}}>0 \end{array}$$

*The supplier’s sustainable green technology innovation effort*

$$\begin{array}{c} \frac{\partial i_{s}^{F2*}}{\partial \lambda }=\frac{\left(A-Bc\right)H_{s}H_{m}k_{s}\Delta_{m}}{{\left[\left(2-3\theta \right)H_{s}\Delta_{m}-\left(1-\theta \right)H_{m}k_{s}^{2}\right]}^{2}}>0 \end{array}$$

This proposition implies that if there is only the manufacturer concerning about the fairness, and the other parameters are kept unchanged, the inequity attitude parameter λ given by the manufacturer will increase and the sustainable green technology innovation development of the manufacturer will provides more efforts. On the other hand, the inequity attitude parameter λ will also increase and the supplier provides more efforts on the sustainable green technology innovation development.

## 5. Numerical Analysis

In this section, in order to obtain the managerial insights on how the utilities and profits of the supply chain members change when fairness concerns vary, following the two cases above, we use numerical experiments to analyze the impacts of fairness concerns on the supplier’s wholesale pricing policy and the manufacturer’s pricing policy, as well as their demands and corresponding sustainable green technology innovation efforts. In addition, we investigated the importance of fairness concerns in their profits and utilities. In our numerical study, the basic parameter values are given as: A=400, B=5, $k_{s}=3$, $k_{m}=2$, $H_{s}=8$, $H_{m}=9$, c=1.

In the first case, if there is only the supplier concerns about fairness, the pricing policy, sustainable green technology innovation efforts, and the utility of the supplier and the manufacturer may suffer significant changes while θ is selected from 0 to 0.5.

**Figure 1:**
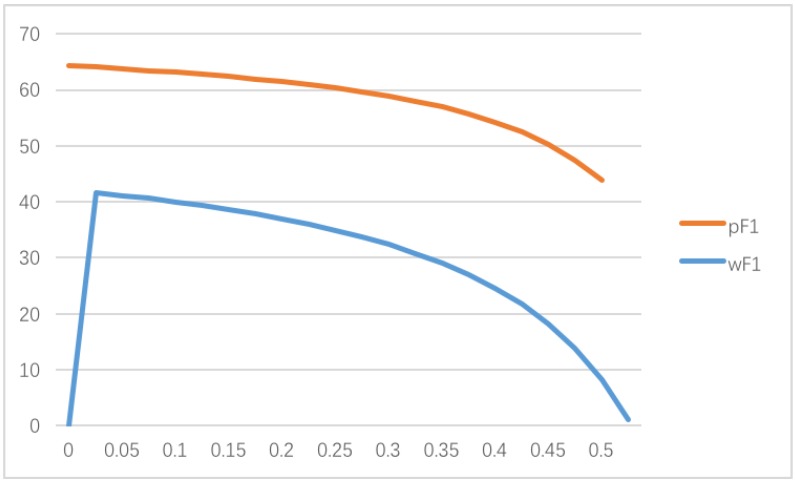
Impact of θ on the pricing policy when only the supplier concerns about fairness.

As shown in Figure 1, in the case only the supplier concerns about fairness, we can find that, starting θ from 0, the supplier’s wholesale price increases dramatically, and, then, slowly drops down.

**Figure 2:**
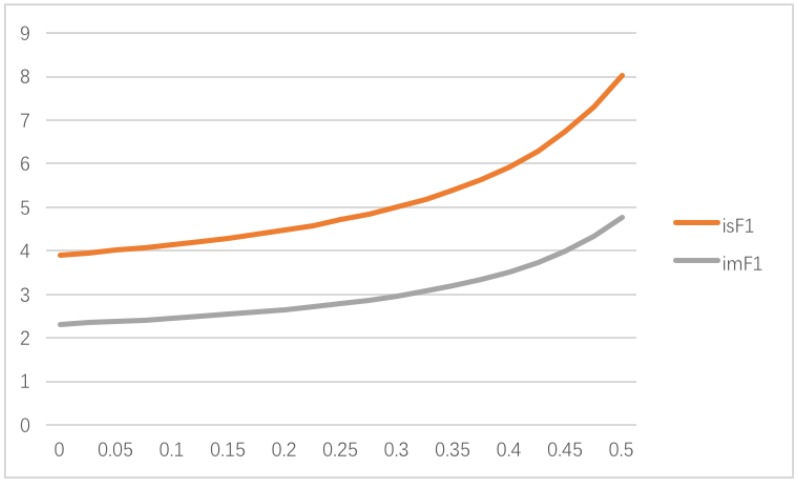
Impact of θ on the sustainable green technology innovation efforts when only the supplier concerns about fairness.

Figure 2 shows the impact of θ on the sustainable green technology innovation efforts when only the supplier concerns about fairness. Since lower θ leads to lower sustainable green technology innovation efforts, the supply chain members may consider improving their sustainable green technology innovation development because it will enhance their sustainable competitiveness in the market. On the other hand, it’s fundamental to keep in mind that higher sustainable green technology innovation efforts also mean that the supply chain members need to invest more financial resources on development, which, of course, may increase their development risk.

**Figure 3:**
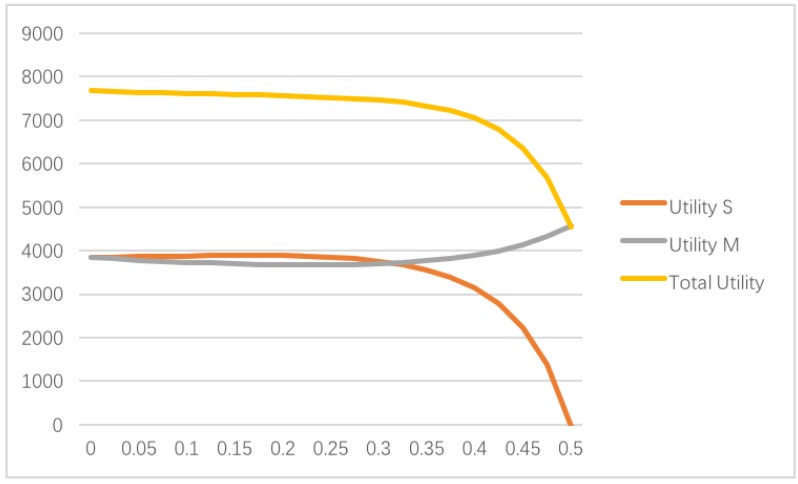
Impact of θ on the utilities when only the supplier concerns about fairness.

It can be seen from Figure 3 that the manufacturer’s utility, which is equal to the manufacturer’s profit, is slightly increasing when only the supplier concerns about fairness. At the same time, the supplier’s utility, as well as the total utility, are decreasing. When θ=0.5, the supplier’s utility drops down to 0, therefore, still from the utility’s point of view, in the Stackelberg model, fairness concerns have a quite important impact on the supplier’s utility when the advantage inequity averseness θ=0.5 gets higher. Compared with the fairness concerns impacts on the supply chain members, the manufacturer is more likely to invest in the sustainable green technology innovation development when θ is increasing. However, the supplier may have less interest in investing in the sustainable green technology innovation development.

If there is only the manufacturer concerns about fairness, the pricing policy, sustainable green technology innovation efforts, and the utility of the supplier and the manufacturer may suffer significant changes when λ is selected from 0 to 0.5.

We conducted a numerical experiment in order to give a better understanding of the impacts of the fairness concerns on the supply chain members’ performances. From the data in Table 2, we can acknowledge that the manufacturer’s selling price $p^{F2*}$ and the supplier’s wholesale price $w^{F2*}$ decrease in λ, which means that the higher the advantage inequity averseness is, the lower the selling price and wholesale price in the supply chain system are. The fairness concerns lead the supply chain members to reduce their prices in the current situation. Therefore, both the supplier and the manufacturer have incentives to pay more attention to their sustainable green technology innovation efforts. However, when λ gets to a higher point, the profit of supplier is negative, which indicates that the supplier has no benefit in the supply chain system when only the manufacturer concerns about fairness.

## 6. Conclusions

In this paper, we develop a quantitative model to evaluate the supply chain system of one supplier and one manufacturer considering fairness concerns with sustainable green technology innovation efforts. We consider this business mode in an operations management horizon which aims to maximize both supplier’s and manufacturer’s utilities. We investigate these objectives under different fairness attitudes in supply chain members. Besides the wholesale and retail price decisions, the optimal sustainable green technology innovation efforts of the supply chain system are also derived and analyzed.

Our major managerial implications are summarized as follows. First, both the supplier and the manufacturer have incentives to join the sustainable green technology innovation development. In fact, when there is only one member (no matter the supplier or the manufacturer) taking part in the development of sustainable green technology innovation, also the other will be interested in joining it, because both of them with the sustainable green technology innovation development could get more benefits (profits or utilities). Second, the fairness concerns is an effective tool used to share the benefits and utilities, which could reduce the potential conflicts of two supply chain members and improve their performances. Third, the strategies of channel members with the fairness concerns could reduce the potential risk of their innovation projects development, and encourage the supply chain members to work together.

### Further Research

In addition, our study holds some limitations that inspire us further potential research opportunities. First, our models assume that the demand functions and the sustainable green technology innovation efforts are linear related. Second, the fairness concerns could also be extended to a more complex setting. Third, the supply chain system with one supplier and one manufacturer could be extended to a system with competitions and other attributes involved.

## Figures and Tables

**Table 1:** Optimal decision results comparison (The benchmarking model of sustainable green technology innovation efforts without fairness concerns by the supplier, by the manufacturer, and by both of them respectively).

	Only Supplier	Only Manufacturer	Both of Them
$p^{*}$	$\frac{6AH_{s}+2BcH_{s}-ck_{s}^{2}}{8BH_{s}-k_{s}^{2}}$	$\frac{2BH_{m}\left(3A+Bc\right)-k_{m}^{2}\left(A+Bc\right)}{2B(4BH_{m}-k_{m}^{2})}$	$\frac{1}{B}\frac{AH_{s}\left(6BH_{m}-k_{m}^{2}\right)+Bc\left(2BH_{s}H_{m}-H_{s}k_{m}^{2}-H_{m}k_{s}^{2}\right)}{2H_{s}\left(4BH_{m}-k_{m}^{2}\right)-H_{m}k_{s}^{2}}$
$w^{*}$	$\frac{4H_{s}\left(A+Bc\right)-ck_{s}^{2}}{8BH_{s}-k_{s}^{2}}$	$\frac{A+Bc}{2B}$	$\frac{1}{B}\frac{H_{s}\left(A+Bc\right)\left(4BH_{m}-k_{m}^{2}\right)-BcH_{m}k_{s}^{2}}{2H_{s}\left(4BH_{m}-k_{m}^{2}\right)-H_{m}k_{s}^{2}}$
$i_{s}^{*}$	$\frac{\left(A-Bc\right)k_{s}}{8BH_{s}-k_{s}^{2}}$	N/A	$\frac{H_{m}k_{s}\left(A-Bc\right)}{2H_{s}\left(4BH_{m}-k_{m}^{2}\right)-H_{m}k_{s}^{2}}$
$i_{m}^{*}$	N/A	$\frac{k_{m}}{2}\frac{A-Bc}{4BH_{m}-k_{m}^{2}}$	$\frac{H_{s}k_{m}\left(A-Bc\right)}{2H_{s}\left(4BH_{m}-k_{m}^{2}\right)-H_{m}k_{s}^{2}}$
$\Pi_{s}^{*}$	$H_{s}\frac{{\left(A-Bc\right)}^{2}}{8BH_{s}-k_{s}^{2}}$	$\frac{H_{m}}{2}\frac{{\left(A-Bc\right)}^{2}}{4BH_{m}-k_{m}^{2}}$	$\frac{H_{m}}{2}\frac{{\left(A-Bc\right)}^{2}}{4BH_{m}-k_{m}^{2}}$
$\Pi_{m}^{*}$	$4BH_{s}^{2}\frac{{\left(A-Bc\right)}^{2}}{{\left(8BH_{s}-k_{s}^{2}\right)}^{2}}$	$\frac{H_{m}}{4}\frac{{\left(A-Bc\right)}^{2}}{4BH_{m}-k_{m}^{2}}$	$\frac{H_{m}}{4}\frac{{\left(A-Bc\right)}^{2}}{4BH_{m}-k_{m}^{2}}$

**Table 2:** Impact of λ when the manufacturer concerns about fairness.

λ	$p^{F2*}$	$w^{F2*}$	$i_{s}^{F2*}$	$i_{m}^{F2*}$	Demand	Profit S	Profit (Utility) M	Utility S
0.00	64.34	41.67	3.90	3.90	36,254.64	1,474,462.59	821,860.18	821,860.18
0.025	64.08	41.16	3.95	3.95	37,242.18	1,495,530.07	853,728.54	837,683.50
0.05	63.80	40.60	4.01	4.01	38,226.27	1,513,841.89	886,817.29	855,466.06
0.075	63.50	40.00	4.07	4.07	39,207.35	1,529,086.14	921,300.61	875,716.69
0.10	63.17	39.34	4.14	4.14	40,185.81	1,540,888.09	957,380.67	899,029.93
0.125	62.81	38.63	4.21	4.21	41,161.97	1,548,797.05	995,294.93	926,107.17
0.15	62.41	37.84	4.29	4.29	42,136.09	1,552,268.86	1,035,325.19	957,783.64
0.175	61.97	36.97	4.38	4.38	43,108.41	1,550,642.86	1,077,809.48	995,063.64
0.20	61.49	36.01	4.48	4.48	44,079.13	1,543,111.01	1,123,157.71	1,039,167.04
0.225	60.95	34.93	4.58	4.58	45,048.43	1,528,676.20	1,171,872.54	1,091,591.71
0.25	60.34	33.73	4.71	4.71	46,016.46	1,506,095.27	1,224,577.99	1,154,198.66
0.275	59.66	32.37	4.85	4.85	46,983.36	1,473,799.59	1,282,058.97	1,229,330.31
0.30	58.88	30.82	5.00	5.00	47,949.27	1,429,782.48	1,345,317.40	1,319,977.87
0.325	57.98	29.04	5.18	5.18	48,914.30	1,371,436.28	1,415,653.22	1,430,023.72
0.35	56.94	26.97	5.39	5.39	49,878.56	1,295,310.32	1,494,784.78	1,564,600.85
0.375	55.71	24.54	5.64	5.64	50,842.18	1,196,741.64	1,585,032.40	1,730,641.43
0.40	54.26	21.64	5.94	5.94	51,805.28	1,069,272.60	1,689,607.75	1,937,741.81
0.425	52.49	18.13	6.29	6.29	52,767.99	903,696.85	1,813,088.05	2,199,579.31
0.45	50.30	13.78	6.74	6.74	53,730.48	686,424.05	1,962,228.57	2,536,340.60
0.475	47.51	8.25	7.30	7.30	54,692.97	396,519.22	2,147,433.85	2,979,118.31
0.50	43.86	1.00	8.04	8.04	55,655.78	−32.30	2,385,607.35	3,578,427.17

## Appendix A. Proofs under Stackelberg Game without Fairness Concerns

**Proof** **of** **Theorem** **1.** We first derive the first partial derivative of the manufacturer’s profit function on $p^{N1}$, which is

(A1) $$\begin{array}{cc} \frac{\partial \Pi_{m}^{N1}}{\partial p^{N1}} & =D\left(p,i_{s}\right)-B\left(p-w\right)\\ & =A-2Bp+k_{s}i_{s}+Bw=0 \end{array}$$

Then we get the expression of $p^{N1}$ with respect to *w* and $i_{s}$,

(A2) $$\begin{array}{cc} p^{N1}\left(w,i_{s}\right) & =\frac{A+k_{s}i_{s}+Bw}{2B} \end{array}$$

We insert $p^{N1}\left(w,i_{s}\right)$ into the demand function, then the supplier’s function (2) should be

(A3) $$\begin{array}{cc} \max_{w,i_{s}}\Pi_{s}^{N1} & =\left(w-c\right)\left(A-\frac{A+k_{s}i_{s}+Bw}{2}+k_{s}i_{s}\right)-H_{s}\cdot i_{s}^{2}\\ & =\frac{A+k_{s}i_{s}-Bw}{2}\left(w-c\right)-H_{s}\cdot i_{s}^{2} \end{array}$$

Next, we derive the first partial derivative of the supplier’s profit function on *w* and $i_{s}$, which means

(A4) $$\left\{\begin{array}{c} \frac{\partial \Pi_{s}^{N1}}{\partial w}=\frac{A+k_{s}i_{s}-Bw}{2}-\left(w-c\right)\frac{B}{2}=\frac{1}{2}\left(A+k_{s}i_{s}-2Bw+Bc\right)=0\\ \frac{\partial \Pi_{s}^{N1}}{\partial i_{s}}=\left(w-c\right)\frac{k_{s}}{2}-2H_{s}i_{s}=\frac{1}{2}\left(k_{s}w-k_{s}c-4H_{s}i_{s}\right)=0 \end{array}\right.$$

or equivalently,

$$\left\{\begin{array}{c} A+k_{s}i_{s}-2Bw+Bc=0\\ k_{s}w-k_{s}c-4H_{s}i_{s}=0 \end{array}\right.$$

From the above equations, we get the optimal unit wholesale price of the supplier $w^{N1*}$, and the optimal sustainable green technology innovation effort of the supplier $i_{s}^{N1*}$.

$$\left\{\begin{array}{c} w^{N1*}=\frac{4H_{s}\left(A+Bc\right)-ck_{s}^{2}}{8BH_{s}-k_{s}^{2}}\\ i_{s}^{N1*}=\frac{A-Bc}{8BH_{s}-k_{s}^{2}}k_{s} \end{array}\right.$$

The objective function of the supplier’s profit above is a linear function of $w^{N1}$ and $i_{s}^{N1}$, which means this objective function is a joint concave function of $w^{N1}$ and $i_{s}^{N1}$. Therefore, we can get the Hessian matrix

$$\left(\begin{array}{cc} \frac{\partial^{2}\Pi_{s}^{N1}}{\partial w^{2}} & \frac{\partial^{2}\Pi_{s}^{N1}}{\partial w\partial i_{s}}\\ \frac{\partial^{2}\Pi_{s}^{N1}}{\partial i_{s}\partial w} & \frac{\partial^{2}\Pi_{s}^{N1}}{\partial i_{s}^{2}} \end{array}\right)=\left(\begin{array}{cc} -B & \frac{k_{s}}{2}\\ \frac{k_{s}}{2} & -2H_{s} \end{array}\right)$$

Then the matrix determinant is

$$\left(-B\right)\left(-2H_{s}\right)-{\left(-\frac{k_{s}}{2}\right)}^{2}=\frac{1}{4}\left(8BH_{s}-k_{s}^{2}\right)>0$$

This Hessian matrix is negative definite, which verified the objective function of the supplier’s profit is a joint concave function of $w^{N1}$ and $i_{s}^{N1}$. In addition, $8BH_{s}-k_{s}^{2}>0$ also holds the assumption. By substituting $w^{N1*}$ and $i_{s}^{N1*}$ to the expression of *p* and demand function, we get the optimal selling price of the manufacturer is $p^{N1*}=\frac{6AH_{s}+2BcH_{s}-ck_{s}^{2}}{8BH_{s}-k_{s}^{2}}$ and the total demand is $D^{N1*}=2BH_{s}\frac{A-Bc}{8BH_{s}-k_{s}^{2}}$. The optimal profit of the supplier is $\Pi_{s}^{N1*}=H_{s}\frac{{\left(A-Bc\right)}^{2}}{8BH_{s}-k_{s}^{2}}$ and the optimal profit of the manufacturer is $\Pi_{m}^{N1*}=4BH_{s}^{2}\frac{{\left(A-Bc\right)}^{2}}{{\left(8BH_{s}-k_{s}^{2}\right)}^{2}}$. ☐

**Proof** **of** **Theorem** **2.** We first derive the first partial derivative of the manufacturer’s profit function on *p* and $i_{m}$, which is

(A5) $$\left\{\begin{array}{cc} \frac{\partial \Pi_{m}^{N2}}{\partial p} & =A-2Bp+k_{m}i_{m}+Bw=0\\ \frac{\partial \Pi_{m}^{N2}}{\partial i_{m}} & =k_{m}\left(p-w\right)-2H_{m}i_{m}=0 \end{array}\right.$$

Then we get the expression of $p^{N2}$ and $i_{m}^{N2}$ with respect to *w*,

$$\left\{\begin{array}{cc} p^{N2}\left(w\right) & =\frac{2AH_{m}+2BH_{m}w-k_{m}^{2}w}{4BH_{m}-k_{m}^{2}}\\ i_{m}^{N2}\left(w\right) & =\frac{\left(A-Bw\right)k_{m}}{4BH_{m}-k_{m}^{2}} \end{array}\right.$$

The above objective function of the manufacturer’s profit is a linear function of $p^{N2}$ and $i_{m}^{N2}$, which means the objective function is a joint concave function of $p^{N2}$ and $i_{m}^{N2}$. Therefore, we can get the Hessian matrix

$$\left(\begin{array}{cc} \frac{\partial^{2}\Pi_{m}^{N2}}{\partial p^{2}} & \frac{\partial^{2}\Pi_{m}^{N2}}{\partial p\partial i_{m}}\\ \frac{\partial^{2}\Pi_{m}^{N2}}{\partial i_{m}\partial p} & \frac{\partial^{2}\Pi_{m}^{N2}}{\partial i_{m}^{2}} \end{array}\right)=\left(\begin{array}{cc} -2B & k_{m}\\ k_{m} & -2H_{m} \end{array}\right)$$

Then the matrix determinant is

$$\left(-2B\right)\left(-2H_{m}\right)-k_{m}^{2}=4BH_{m}-k_{m}^{2}>0$$

This Hessian matrix is negative definite, which verified the objective function of the manufacturer’s profit is a joint concave function of $p^{N2}$ and $i_{m}^{N2}$. In addition, $4BH_{m}-k_{m}^{2}>0$ also holds the assumption. We insert $p^{N2}\left(w\right)$ and $i_{m}^{N2}\left(w\right)$ into the demand function, then the supplier’s function (4) should be

(A6) $$\begin{array}{cc} \max_{w^{N2}}\Pi_{s}^{N2} & =\left(w-c\right)\left(A-B\frac{2AH_{m}+2BH_{m}w-k_{m}^{2}w}{4BH_{m}-k_{m}^{2}}+k_{m}\frac{\left(A-Bw\right)k_{m}}{4BH_{m}-k_{m}^{2}}\right)\\ & =2BH_{m}\left(w-c\right)\frac{A-Bw}{4BH_{m}-k_{m}^{2}} \end{array}$$

The first partial derivative on $w^{N2}$, we get

(A7) $$\begin{array}{cc} \frac{\partial \Pi_{s}^{N2}}{\partial w^{N2}} & =2BH_{m}\frac{\left(A-Bw)-B(w-c\right)}{4BH_{m}-k_{m}^{2}}\\ & =2BH_{m}\frac{A+Bc-2Bw}{4BH_{m}-k_{m}^{2}}=0 \end{array}$$

Then we get the optimal unit wholesale price of the supplier

(A8) $$\begin{array}{c} w^{N2*}=\frac{A+Bc}{2B} \end{array}$$

By substituting the optimal $w^{N2*}$ to the expression of $p^{N2}$ and $i_{m}^{N2}$ and the demand function $D^{N2}$, we get the optimal selling price of the manufacturer, $p^{N2*}=\frac{2BH_{m}\left(3A+Bc\right)-k_{m}^{2}\left(A+Bc\right)}{2B(4BH_{m}-k_{m}^{2})}$, the optimal sustainable green technology innovation effort of the manufacturer, $i_{m}^{N2*}=\frac{k_{m}}{2}\frac{A-Bc}{4BH_{m}-k_{m}^{2}}$, and the total demand, $D^{N2*}=BH_{m}\frac{A-Bc}{4BH_{m}-k_{m}^{2}}$. The optimal profit of the supplier is $\Pi_{s}^{N2*}=\frac{H_{m}}{2}\frac{{\left(A-Bc\right)}^{2}}{4BH_{m}-k_{m}^{2}}$ and the optimal profit of the manufacturer is $\Pi_{m}^{N2*}=\frac{H_{m}}{4}\frac{{\left(A-Bc\right)}^{2}}{4BH_{m}-k_{m}^{2}}$. ☐

**Proof** **of** **Theorem** **3.** We first derive the first partial derivative of the manufacturer’s profit function on $p^{N3}$ and $i_{m}^{N3}$, which is

(A9) $$\left\{\begin{array}{cc} \frac{\partial \Pi_{m}^{N3}}{\partial p} & =A-2Bp+k_{s}i_{s}+k_{m}i_{m}+Bw=0\\ \frac{\partial \Pi_{m}^{N3}}{\partial i_{m}} & =\left(p-w\right)k_{m}-2H_{m}i_{m}=0 \end{array}\right.$$

Then we get

$$\left\{\begin{array}{cc} p^{N3}\left(w,i_{s}\right) & =\frac{2AH_{m}+2H_{m}k_{s}i_{s}+2BH_{m}w-k_{m}^{2}w}{4BH_{m}-k_{m}^{2}}\\ i_{m}^{N3}\left(w,i_{s}\right) & =k_{m}\frac{A+k_{s}i_{s}-Bw}{4BH_{m}-k_{m}^{2}} \end{array}\right.$$

The objective function of the manufacturer’s profit above is a linear function of $p^{N3}$ and $i_{m}^{N3}$, which means this objective function is a joint concave function of $p^{N3}$ and $i_{m}^{N3}$. Therefore, we can get the Hessian matrix

$$\left(\begin{array}{cc} \frac{\partial^{2}\Pi_{m}^{N3}}{\partial p^{2}} & \frac{\partial^{2}\Pi_{m}^{N3}}{\partial p\partial i_{m}}\\ \frac{\partial^{2}\Pi_{m}^{N3}}{\partial p\partial i_{m}} & \frac{\partial^{2}\Pi_{m}^{N3}}{\partial i_{m}^{2}} \end{array}\right)=\left(\begin{array}{cc} -2B & k_{m}\\ k_{m} & -2H_{m} \end{array}\right)$$

Then the matrix determinant is

$$\left(-2B\right)\left(-2H_{m}\right)-k_{m}^{2}=4BH_{m}-k_{m}^{2}>0$$

This Hessian matrix is negative definite, which verified the objective function of the manufacturer’s profit is a joint concave function of $p^{N3}$ and $i_{m}^{N3}$. In addition, $4BH_{m}-k_{m}^{2}>0$ also holds the assumption. When we insert $p^{N3}\left(w,i_{s}\right)$ and $i_{m}^{N3}\left(w,i_{s}\right)$ into the demand function, the supplier’s function (6) should be

(A10) $$\begin{array}{c} \max_{w^{N3},i_{s}^{N3}}\Pi_{s}^{N3}=\left(w-c\right)\frac{2BH_{m}\left(A-Bw+k_{s}i_{s}\right)}{4BH_{m}-k_{m}^{2}}-H_{s}\cdot i_{s}^{2} \end{array}$$

The first partial derivative on $w^{N3}$ and $i_{s}^{N3}$, we get

(A11) $$\left\{\begin{array}{c} \frac{\partial \Pi_{s}^{N3}}{\partial w}=\frac{2BH_{m}\left(A-Bw+k_{s}i_{s}\right)}{4BH_{m}-k_{m}^{2}}-\left(w-c\right)\frac{2B^{2}H_{m}}{4BH_{m}-k_{m}^{2}}=0\\ \frac{\partial \Pi_{s}^{N3}}{\partial i_{s}}=\left(w-c\right)\frac{2k_{s}BH_{m}}{4BH_{m}-k_{m}^{2}}-2H_{s}i_{s}=0 \end{array}\right.$$

The optimal $w^{N3*}$ and $i_{s}^{N3*}$ are listing below,

$$\left\{\begin{array}{c} w^{N3*}=\frac{1}{B}\frac{H_{s}\left(A+Bc\right)\left(4BH_{m}-k_{m}^{2}\right)-BcH_{m}k_{s}^{2}}{2H_{s}\left(4BH_{m}-k_{m}^{2}\right)-H_{m}k_{s}^{2}}\\ i_{s}^{N3*}=\frac{H_{m}k_{s}\left(A-Bc\right)}{2H_{s}\left(4BH_{m}-k_{m}^{2}\right)-H_{m}k_{s}^{2}} \end{array}\right.$$

The objective function of the supplier’s profit above is a linear function of $w^{N3}$ and $i_{s}^{N3}$, which means this objective function is a joint concave function of $w^{N3}$ and $i_{s}^{N3}$. Therefore, we can get the Hessian matrix

$$\left(\begin{array}{cc} \frac{\partial^{2}\Pi_{m}^{N3}}{\partial w^{2}} & \frac{\partial^{2}\Pi_{m}^{N2}}{\partial w\partial i_{s}}\\ \frac{\partial^{2}\Pi_{m}^{N3}}{\partial i_{s}\partial w} & \frac{\partial^{2}\Pi_{m}^{N3}}{\partial i_{s}^{2}} \end{array}\right)=\left(\begin{array}{cc} \frac{-4B^{2}H_{m}}{4BH_{m}-k_{m}^{2}} & \frac{2BH_{m}k_{s}}{4BH_{m}-k_{m}^{2}}\\ \frac{2BH_{m}k_{s}}{4BH_{m}-k_{m}^{2}} & -2H_{s} \end{array}\right)$$

Then the matrix determinant is

$$\frac{8B^{2}H_{s}H_{m}}{4BH_{m}-k_{m}^{2}}-{\left(\frac{2BH_{m}k_{s}}{4BH_{m}-k_{m}^{2}}\right)}^{2}=\frac{4B^{2}H_{m}}{4BH_{m}-k_{m}^{2}}\left[2H_{s}\left(4BH_{m}-k_{m}^{2}\right)-H_{m}k_{s}^{2}\right]>0$$

Therefore, this Hessian matrix is negative definite, which verified the objective function of the supplier’s profit is a joint concave function of *w* and $i_{s}$.From the supplier’s matrix determinant we know $4BH_{m}-k_{m}^{2}>0$, then $2H_{s}\left(4BH_{m}-k_{m}^{2}\right)-H_{m}k_{s}^{2}>0$ also holds the assumption. We insert the optimal $w^{N3*}$ and $i_{s}^{N3*}$ into $p^{N3}\left(w,i_{s}\right)$ and $i_{m}^{N3}\left(w,i_{s}\right)$, so we get the optimal selling price of the manufacturer $p^{N3*}=\frac{1}{B}\frac{AH_{s}\left(6BH_{m}-k_{m}^{2}\right)+Bc\left(2BH_{s}H_{m}-H_{s}k_{m}^{2}-H_{m}k_{s}^{2}\right)}{2H_{s}\left(4BH_{m}-k_{m}^{2}\right)-H_{m}k_{s}^{2}}$, the optimal sustainable green technology innovation effort of the manufacturer $i_{m}^{N3*}=\frac{H_{m}k_{s}\left(A-Bc\right)}{2H_{s}\left(4BH_{m}-k_{m}^{2}\right)-H_{m}k_{s}^{2}}$, and the total demand $D^{N3*}=BH_{m}\frac{A-Bc}{4BH_{m}-k_{m}^{2}}$. The optimal profit of the supplier is $\Pi_{s}^{N3*}=\frac{H_{m}}{2}\frac{{\left(A-Bc\right)}^{2}}{4BH_{m}-k_{m}^{2}}$ and the optimal profit of the manufacturer is $\Pi_{m}^{N3*}=\frac{H_{m}}{4}\frac{{\left(A-Bc\right)}^{2}}{4BH_{m}-k_{m}^{2}}$. ☐

**Proof** **of** **Proposition** **1.** Comparing the profit values of the supplier and the manufacturer with and without sustainable green technology innovation efforts, we can get the profits difference as the following:

(A12) $$\Pi_{s}^{N1*}-\Pi_{s}^{0*}=H_{s}\frac{{\left(A-Bc\right)}^{2}}{8BH_{s}-k_{s}^{2}}-\frac{{\left(A-Bc\right)}^{2}}{8B}$$

(A13) $$={\left(A-Bc\right)}^{2}\frac{k_{s}^{2}}{8B(8BH_{s}-k_{s}^{2})}$$

We already assumed that $8BH_{s}-k_{s}^{2}>0$, therefore we get $\Pi_{s}^{N1*}>\Pi_{s}^{0*}$.

(A14) $$\Pi_{s}^{N2*}-\Pi_{s}^{0*}=\frac{H_{m}}{2}\frac{{\left(A-Bc\right)}^{2}}{4BH_{m}-k_{m}^{2}}-\frac{{\left(A-Bc\right)}^{2}}{8B}$$

(A15) $$=\frac{{\left(A-Bc\right)}^{2}}{8B(4BH_{m}-k_{m}^{2})}\cdot k_{m}^{2}$$

We already assumed that $4BH_{m}-k_{m}^{2}>0$, therefore we get $\Pi_{s}^{N2*}>\Pi_{s}^{0*}$.

(A16) $$\Pi_{m}^{N1*}-\Pi_{m}^{0*}=4BH_{s}^{2}\frac{{\left(A-Bc\right)}^{2}}{{\left(8BH_{s}-k_{s}^{2}\right)}^{2}}-\frac{{\left(A-Bc\right)}^{2}}{16B}$$

(A17) $$=\frac{{\left(A-Bc\right)}^{2}}{16B{\left(8BH_{s}-k_{s}^{2}\right)}^{2}}\cdot k_{s}^{2}\cdot \left(16BH_{s}-k_{s}^{2}\right)$$

We already assumed that $8BH_{s}-k_{s}^{2}>0$, then $16BH_{s}-k_{s}^{2}>8BH_{s}-k_{s}^{2}>0$, therefore we get $\Pi_{m}^{N1*}>\Pi_{m}^{0*}$.

$$\begin{array}{cc} \Pi_{m}^{N2*}-\Pi_{m}^{0*}= & \frac{H_{m}}{4}\frac{{\left(A-Bc\right)}^{2}}{4BH_{m}-k_{m}^{2}}-\frac{{\left(A-Bc\right)}^{2}}{16B}\\ = & \frac{{\left(A-Bc\right)}^{2}}{16B(4BH_{m}-k_{m}^{2})}\left(4BH_{m}-\left(4BH_{m}-k_{m}^{2}\right)\right) \end{array}$$

We already assumed that $4BH_{m}-k_{m}^{2}>0$, therefore we get $\Pi_{m}^{N2*}>\Pi_{m}^{0*}$. ☐

## Appendix B. Proofs under Stackelberg Game with Fairness Concerns

**Proof** **of** **Theorem** **4.** At first, we derive the first partial derivative of the manufacturer’s utility function $\mu (\Pi_{m})$ on *p* and $i_{m}$, which is

(A18) $$\left\{\begin{array}{cc} \frac{\partial \mu^{F1}\left(\Pi_{m}\right)}{\partial p} & =A-2Bp+k_{s}i_{s}+k_{m}i_{m}+Bw=0\\ \frac{\partial \mu^{F1}\left(\Pi_{m}\right)}{\partial i_{m}} & =\left(p-w\right)k_{m}-2H_{m}i_{m}=0 \end{array}\right.$$

Then we get

$$\left\{\begin{array}{cc} p^{F1}\left(w,i_{s}\right) & =\frac{2AH_{m}+2H_{m}k_{s}i_{s}+2BH_{m}w-k_{m}^{2}w}{4BH_{m}-k_{m}^{2}}\\ i_{m}^{F1}\left(w,i_{s}\right) & =k_{m}\frac{A+k_{s}i_{s}-Bw}{4BH_{m}-k_{m}^{2}} \end{array}\right.$$

The objective function of the manufacturer’s utility above is a linear function of *p* and $i_{m}$, which means this objective function is a joint concave function of *p* and $i_{m}$. Therefore, we can get the Hessian matrix

$$\left(\begin{array}{cc} \frac{\partial^{2}\mu^{F1}\left(\Pi_{m}\right)}{\partial p^{2}} & \frac{\partial^{2}\mu^{F1}\left(\Pi_{m}\right)}{\partial p\partial i_{m}}\\ \frac{\partial^{2}\mu^{F1}\left(\Pi_{m}\right)}{\partial i_{m}\partial p} & \frac{\partial^{2}\mu^{F1}\left(\Pi_{m}\right)}{\partial i_{m}^{2}} \end{array}\right)=\left(\begin{array}{cc} -2B & k_{m}\\ k_{m} & -2H_{m} \end{array}\right)$$

Then the matrix determinant is

$$\left(-2B\right)\left(-2H_{m}\right)-k_{m}^{2}=4BH_{m}-k_{m}^{2}>0$$

We assume $\Delta_{m}=4BH_{m}-k_{m}^{2}$. This Hessian matrix is negative definite, which verified the objective function of the manufacturer’s profit is a joint concave function of $p^{F1}$ and $i_{m}^{F1}$. In addition, $4BH_{m}-k_{m}^{2}>0$ also holds the assumption. We insert $p^{F1}\left(w,i_{s}\right)$ and $i_{m}^{F1}\left(w,i_{s}\right)$ into the demand function,

(A19) $$\begin{array}{c} D^{F1}\left(w,i_{s}\right)=\frac{2BH_{m}\left(A-Bw+k_{s}i_{s}\right)}{4BH_{m}-k_{m}^{2}} \end{array}$$

Then the supplier’s utility function (9) should be

$$\begin{array}{ccc} \max_{w^{F1},i_{s}^{F1}}\mu^{F1}\left(\Pi_{s}\right) & = & \left(1-\theta \right)\Pi_{s}+\theta \Pi_{m}\\ & = & \left(1-\theta \right)\left(w-c\right)\frac{2BH_{m}\left(A-Bw+k_{s}i_{s}\right)}{4BH_{m}-k_{m}^{2}}-\left(1-\theta \right)H_{s}\cdot i_{s}^{2}\\ & & +\theta \left(p-w\right)\frac{2BH_{m}\left(A-Bw+k_{s}i_{s}\right)}{4BH_{m}-k_{m}^{2}}-\theta H_{m}\cdot i_{m}^{2}\\ & = & \frac{\left(1-\theta \right)2BH_{m}}{4BH_{m}-k_{m}^{2}}\left(w-c\right)\left(A-Bw+k_{s}i_{s}\right)-\left(1-\theta \right)H_{s}\cdot i_{s}^{2}+\frac{\theta H_{m}}{4BH_{m}-k_{m}^{2}}{\left(A-Bw+k_{s}i_{s}\right)}^{2} \end{array}$$

The first partial derivative on $w^{F1}$ and $i_{s}^{F1}$, we get

$$\left\{\begin{array}{cc} \frac{\partial \mu^{F1}\left(\Pi_{s}\right)}{\partial i_{s}}= & 0\\ \frac{\partial \mu^{F1}\left(\Pi_{s}\right)}{\partial w}= & 0 \end{array}\right.$$

under which,

$$\begin{array}{cc} \frac{\partial \mu^{F1}\left(\Pi_{s}\right)}{\partial i_{s}}= & \frac{\left(1-\theta \right)2BH_{m}k_{s}}{4BH_{m}-k_{m}^{2}}\left(w-c\right)-2\left(1-\theta \right)H_{s}\cdot i_{s}+\frac{2k_{s}\theta H_{m}}{4BH_{m}-k_{m}^{2}}\left(A-Bw+k_{s}i_{s}\right)\\ = & \frac{2}{\Delta_{m}}\left[\left(1-\theta \right)BH_{m}k_{s}\left(w-c\right)-\left(1-\theta \right)H_{s}\cdot i_{s}\Delta_{m}+k_{s}\theta H_{m}\left(A-Bw+k_{s}i_{s}\right)\right] \end{array}$$

$$\begin{array}{cc} \frac{\partial \mu^{F1}\left(\Pi_{s}\right)}{\partial w}= & \frac{\left(1-\theta \right)2BH_{m}}{4BH_{m}-k_{m}^{2}}\left(A-2Bw+k_{s}i_{s}+Bc\right)-2B\frac{\theta H_{m}}{4BH_{m}-k_{m}^{2}}\left(A-Bw+k_{s}i_{s}\right)\\ = & \frac{2BH_{m}}{4BH_{m}-k_{m}^{2}}\left[\left(1-\theta \right)\left(A-2Bw+k_{s}i_{s}+Bc\right)-\theta \left(A-Bw+k_{s}i_{s}\right)\right] \end{array}$$

The optimal $w^{F1*}$ and $i_{s}^{F1*}$ are listed below,

$$\left\{\begin{array}{c} w^{F1*}=\frac{1}{B}\frac{\left(1-2\theta \right)AH_{s}\Delta_{m}+\left(1-\theta \right)BcH_{s}\Delta_{m}-\left(1-\theta \right)BcH_{m}k_{s}^{2}}{\left(2-3\theta \right)H_{s}\Delta_{m}-\left(1-\theta \right)H_{m}k_{s}^{2}}\\ i_{s}^{F1*}=\frac{\left(1-\theta \right)H_{m}k_{s}\left(A-Bc\right)}{\left(2-3\theta \right)H_{s}\Delta_{m}-\left(1-\theta \right)H_{m}k_{s}^{2}} \end{array}\right.$$

The objective function of the supplier’s profit above is a linear function of $w^{F1*}$ and $i_{s}^{F1*}$, which means this objective function is a joint concave function of $w^{F1*}$ and $i_{s}^{F1*}$. Therefore, we can get the Hessian matrix

$$\left(\begin{array}{cc} \frac{\partial^{2}\mu \left(\Pi_{s}\right)}{\partial w^{2}} & \frac{\partial^{2}\mu \left(\Pi_{s}\right)}{\partial w\partial i_{s}}\\ \frac{\partial^{2}\mu \left(\Pi_{s}\right)}{\partial i_{s}\partial w} & \frac{\partial^{2}\mu \left(\Pi_{s}\right)}{\partial i_{s}^{2}} \end{array}\right)=\left(\begin{array}{cc} \frac{-2\left(2-3\theta \right)B^{2}H_{m}}{\Delta_{m}} & \frac{2\left(1-2\theta \right)BH_{m}k_{s}}{\Delta_{m}}\\ \frac{2\left(1-2\theta \right)BH_{m}k_{s}}{\Delta_{m}} & \frac{-2[\left(1-\theta \right)H_{s}\Delta_{m}-\theta k_{s}^{2}H_{m}]}{\Delta_{m}} \end{array}\right)$$

Then the matrix determinant is

$$\begin{array}{c} \;\frac{4\left(2-3\theta \right)B^{2}H_{m}\left[\left(1-\theta \right)H_{s}\Delta_{m}-\theta k_{s}^{2}H_{m}\right]}{\Delta_{m}^{2}}-{\left(\frac{2\left(1-2\theta \right)BH_{m}k_{s}}{\Delta_{m}}\right)}^{2}\\ =\frac{4\left(1-\theta \right)B^{2}H_{m}}{\Delta_{m}^{2}}\left[\left(2-3\theta \right)H_{s}\Delta_{m}-\left(1-\theta \right)H_{m}k_{s}^{2}\right]>0 \end{array}$$

Therefore, this Hessian matrix is negative definite, which verified the objective function of the supplier’s profit is a joint concave function of *w* and $i_{s}$. From the supplier’s matrix determinant we know $\Delta_{m}=4BH_{m}-k_{m}^{2}>0$, then $\left(2-3\theta \right)H_{s}\Delta_{m}-\left(1-\theta \right)H_{m}k_{s}^{2}>0$ also holds the assumption. We insert the optimal $w^{F1*}$ and $i_{s}^{F1*}$ into $p(w,i_{s})$ and $i_{m}\left(w,i_{s}\right)$, then we get the optimal selling price of the manufacturer

$$\begin{array}{c} p^{F1*}=\frac{1}{B}\frac{AH_{s}\left[2\left(3-5\theta \right)BH_{m}+\left(1-2\theta \right)k_{m}^{2}\right]+Bc\left(1-\theta \right)\left[2BH_{m}H_{s}-H_{s}k_{m}^{2}-H_{m}k_{s}^{2}\right]}{\left(2-3\theta \right)H_{s}\Delta_{m}-\left(1-\theta \right)H_{m}k_{s}^{2}} \end{array}$$

and the optimal sustainable green technology innovation effort of the manufacturer

$$i_{m}^{F1*}=\frac{\left(1-\theta \right)\left(A-Bc\right)H_{s}k_{m}}{\left(2-3\theta \right)H_{s}\Delta_{m}-\left(1-\theta \right)H_{m}k_{s}^{2}}$$

and the total demand is $D^{F1*}=\frac{2\left(1-\theta \right)B\left(A-Bc\right)H_{m}H_{s}}{\left(2-3\theta \right)H_{s}\Delta_{m}-\left(1-\theta \right)H_{m}k_{s}^{2}}$. The optimal profit of the supplier is

$$\Pi_{s}^{F1*}=\frac{A-Bc}{B}\frac{\left(1-2\theta \right)\left(2-3\theta \right)H_{s}^{2}\Delta_{m}^{2}-\left(1-\theta \right)\left(1-2\theta \right)H_{m}H_{s}k_{s}^{2}\Delta_{m}-{\left(1-\theta \right)}^{2}BH_{m}^{2}H_{s}k_{s}^{2}\left(A-Bc\right)}{{\left[\left(2-3\theta \right)H_{s}\Delta_{m}-\left(1-\theta \right)H_{m}k_{s}^{2}\right]}^{2}}$$

and the optimal profit of the manufacturer is

$$\Pi_{m}^{F1*}=\left(1-\theta \right)\left(A-Bc\right)H_{m}\frac{\begin{array}{c} 4\left(5-9\theta \right)ABH_{m}H_{s}^{2}-4\left(1-\theta \right)B^{2}cH_{m}H_{s}^{2}\\ -4\left(1-2\theta \right)A\Delta_{m}H_{s}^{2}-\left(1-\theta \right)\left(A-Bc\right)H_{m}^{2}k_{s}^{2} \end{array}}{{\left[\left(2-3\theta \right)H_{s}\Delta_{m}-\left(1-\theta \right)H_{m}k_{s}^{2}\right]}^{2}}$$

$$\begin{array}{cc} \mu^{F1*}\left(\Pi_{s}\right)= & \left(1-\theta \right)\Pi_{s}^{F1*}+\theta \Pi_{m}^{F1*}\\ = & \frac{\left(1-\theta )(A-Bc\right)}{B{\left[\left(2-3\theta \right)H_{s}\Delta_{m}-\left(1-\theta \right)H_{m}k_{s}^{2}\right]}^{2}}\left[\cdot \right] \end{array}$$

Where the expression of [·] is

$$\begin{array}{c} \left[\cdot \right]=\left(1-2\theta \right)\left(2-3\theta \right)H_{s}^{2}\Delta_{m}^{2}-\left(1-\theta \right)\left(1-2\theta \right)H_{m}H_{s}k_{s}^{2}\Delta_{m}-{\left(1-\theta \right)}^{2}BH_{m}^{2}H_{s}k_{s}^{2}\left(A-Bc\right)\\ +4\left(5-9\theta \right)\theta AB^{2}H_{m}^{2}H_{s}^{2}-4\left(1-\theta \right)\theta B^{3}cH_{m}^{2}H_{s}^{2}-4\left(1-2\theta \right)\theta AB\Delta_{m}H_{m}H_{s}^{2}-\left(1-\theta \right)\theta \left(A-Bc\right)BH_{m}^{3}k_{s}^{2} \end{array}$$

 ☐

**Proof** **of** **Proposition** **2.** If there is only the supplier concerning about fairness, The manufacturer’s sustainable green technology innovation effort is

(A20) $$\frac{\partial i_{m}^{F1*}}{\partial \theta }=\frac{\left\{\begin{array}{c} -\left(A-Bc\right)H_{s}k_{m}\left[\left(2-3\theta \right)H_{s}\Delta_{m}-\left(1-\theta \right)H_{m}k_{s}^{2}\right]\\ -\left(-3H_{s}\Delta_{m}+H_{m}k_{s}^{2}\right)\left[\left(1-\theta \right)\left(A-Bc\right)H_{s}k_{m}\right] \end{array}\right\}}{{\left[\left(2-3\theta \right)H_{s}\Delta_{m}-\left(1-\theta \right)H_{m}k_{s}^{2}\right]}^{2}}$$

(A21) $$=\frac{\left(A-Bc\right)H_{s}^{2}k_{m}\Delta_{m}}{{\left[\left(2-3\theta \right)H_{s}\Delta_{m}-\left(1-\theta \right)H_{m}k_{s}^{2}\right]}^{2}}>0$$

The supplier’s sustainable green technology innovation effort is

(A22) $$\frac{\partial i_{s}^{F1*}}{\partial \theta }=\frac{\left\{\begin{array}{c} -\left(A-Bc\right)H_{m}k_{s}\left[\left(2-3\theta \right)H_{s}\Delta_{m}-\left(1-\theta \right)H_{m}k_{s}^{2}\right]\\ -\left(-3H_{s}\Delta_{m}+H_{m}k_{s}^{2}\right)\left[\left(1-\theta \right)\left(A-Bc\right)H_{m}k_{s}\right] \end{array}\right\}}{{\left[\left(2-3\theta \right)H_{s}\Delta_{m}-\left(1-\theta \right)H_{m}k_{s}^{2}\right]}^{2}}$$

(A23) $$=\frac{\left(A-Bc\right)H_{s}H_{m}k_{s}\Delta_{m}}{{\left[\left(2-3\theta \right)H_{s}\Delta_{m}-\left(1-\theta \right)H_{m}k_{s}^{2}\right]}^{2}}>0$$

 ☐

**Proof** **of** **Theorem** **5.** At first, we derive the first partial derivative of the manufacturer’s utility function $\mu (\Pi_{m})$ on $p^{F2}$ and $i_{m}^{F2}$, which is

$$\left\{\begin{array}{cc} \frac{\partial \mu^{F2}\left(\Pi_{m}\right)}{\partial p} & =\left(1+\lambda \right)\left(A-2Bp+k_{s}i_{s}+k_{m}i_{m}+Bw\right)-\lambda \left(-B\right)\left(w-c\right)=0\\ \frac{\partial \mu^{F2}\left(\Pi_{m}\right)}{\partial i_{m}} & =\left(1+\lambda \right)\left[\left(p-w\right)k_{m}-2H_{m}i_{m}\right]-\lambda k_{m}\left(w-c\right)=0 \end{array}\right.$$

Then we get

$$\left\{\begin{array}{cc} p^{F2}\left(w,i_{s}\right) & =\frac{\left(1+2\lambda \right)\left(2BH_{m}-k_{m}^{2}\right)w+\lambda \left(k_{m}^{2}-2BH_{m}\right)c+2\left(1+\lambda \right)H_{m}\left(A+k_{s}i_{s}\right)}{\left(1+\lambda \right)\Delta_{m}}\\ i_{m}^{F2}\left(w,i_{s}\right) & =k_{m}\frac{\left(1+\lambda \right)\left(A+k_{s}i_{s}\right)-\left(1+2\lambda \right)Bw+\lambda Bc}{\left(1+\lambda \right)\Delta_{m}} \end{array}\right.$$

The objective function of the manufacturer’s utility above is a linear function of $p^{F2}$ and $i_{m}^{F2}$, which means this objective function is a joint concave function of $p^{F2}$ and $i_{m}^{F2}$. Therefore, we can get the Hessian matrix

$$\left(\begin{array}{cc} \frac{\partial^{2}\mu \left(\Pi_{m}\right)}{\partial p^{2}} & \frac{\partial^{2}\mu \left(\Pi_{m}\right)}{\partial p\partial i_{m}}\\ \frac{\partial^{2}\mu \left(\Pi_{m}\right)}{\partial p\partial i_{m}} & \frac{\partial^{2}\mu \left(\Pi_{m}\right)}{\partial i_{m}^{2}} \end{array}\right)=\left(\begin{array}{cc} -2B(1+\lambda ) & \left(1+\lambda \right)k_{m}\\ \left(1+\lambda \right)k_{m} & -2H_{m}\left(1+\lambda \right) \end{array}\right)$$

Then the matrix determinant is

$$\begin{array}{ccc} \left(-2B\right)\left(-2H_{m}\right){\left(1+\lambda \right)}^{2}-{\left(1+\lambda \right)}^{2}k_{m}^{2} & = & {\left(1+\lambda \right)}^{2}\left(4BH_{m}-k_{m}^{2}\right)\\ & = & {\left(1+\lambda \right)}^{2}\Delta_{m}>0 \end{array}$$

This Hessian matrix is negative definite, which verified the objective function of the manufacturer’s profit is a joint concave function of $p^{F2}$ and $i_{m}^{F2}$. In addition, ${\left(1+\lambda \right)}^{2}\Delta_{m}>0$ also holds the assumption. We insert $p^{F2}\left(w,i_{s}\right)$ and $i_{m}^{F2}\left(w,i_{s}\right)$ into the demand function,

$$\begin{array}{c} D^{F2}\left(p,i_{s},i_{m}\right)=\frac{2B}{\left(1+\lambda \right)\Delta_{m}}\left[\left(1+\lambda \right)H_{m}\left(A+k_{s}i_{s}\right)+\lambda \left(k_{m}^{2}-BH_{m}\right)c+\left(1+2\lambda \right)\left(BH_{m}-k_{m}^{2}\right)w\right] \end{array}$$

Then the supplier’s utility function (15) should be

$$\begin{array}{ccc} \max_{w,i_{s}}\mu^{F2}\left(\Pi_{s}\right) & = & \frac{2B(w-c)}{\left(1+\lambda \right)\Delta_{m}}[\left(1+\lambda \right)H_{m}\left(A+k_{s}i_{s}\right)+\lambda \left(k_{m}^{2}-BH_{m}\right)c\\ & & +\left(1+2\lambda \right)\left(BH_{m}-k_{m}^{2}\right)w]-H_{s}\cdot i_{s}^{2} \end{array}$$

The first partial derivative on *w* and $i_{s}$, we get

$$\left\{\begin{array}{cc} \frac{\partial \mu^{F2}\left(\Pi_{s}\right)}{\partial w}= & \frac{2B}{\left(1+\lambda \right)\Delta_{m}}\left\{\left(1+\lambda \right)H_{m}\left(A+k_{s}i_{s}\right)-\left(1+\lambda \right)\left(BH_{m}-k_{m}^{2}\right)c+2\left(1+2\lambda \right)\left(BH_{m}-k_{m}^{2}\right)w\right\}=0\\ \frac{\partial \mu^{F2}\left(\Pi_{s}\right)}{\partial i_{s}}= & \frac{2BH_{m}k_{s}}{\Delta_{m}}\left(w-c\right)-2H_{s}i_{s}=0 \end{array}\right.$$

Under which, $i_{s}^{F2}=\frac{BH_{m}k_{s}}{H_{s}\Delta_{m}}\left(w-c\right)$, substitute it back, we can get

$$\begin{array}{c} w^{F2*}=\frac{\left(1+\lambda \right)\left[\left(BH_{m}-k_{m}^{2}\right)H_{s}\Delta_{m}+BH_{m}^{2}k_{s}^{2}\right]c+\left(1+\lambda \right)AH_{m}H_{s}\Delta_{m}}{2\left(1+2\lambda \right)\left(BH_{m}-k_{m}^{2}\right)H_{s}\Delta_{m}+\left(1+\lambda \right)BH_{m}^{2}k_{s}^{2}} \end{array}$$

Therefore, we can easily get

$$\begin{array}{cc} i_{s}^{F2*}= & \frac{BH_{m}k_{s}}{H_{s}\Delta_{m}}\left(\frac{\left(1+\lambda \right)\left[\left(BH_{m}-k_{m}^{2}\right)H_{s}\Delta_{m}+BH_{m}^{2}k_{s}^{2}\right]c+\left(1+\lambda \right)AH_{m}H_{s}\Delta_{m}}{2\left(1+2\lambda \right)\left(BH_{m}-k_{m}^{2}\right)H_{s}\Delta_{m}+\left(1+\lambda \right)BH_{m}^{2}k_{s}^{2}}-c\right)\\ = & BH_{m}k_{s}\frac{\left(1+\lambda \right)AH_{m}-\left(1+3\lambda \right)\left(BH_{m}-k_{m}^{2}\right)c}{2\left(1+2\lambda \right)\left(BH_{m}-k_{m}^{2}\right)H_{s}\Delta_{m}+\left(1+\lambda \right)BH_{m}^{2}k_{s}^{2}} \end{array}$$

The objective function of the supplier’s utility above is a linear function of $w^{F2}$ and $i_{s}^{F2}$, which means this objective function is a joint concave function of $w^{F2}$ and $i_{s}^{F2}$. Therefore, we can get the Hessian matrix

$$\left(\begin{array}{cc} \frac{\partial^{2}\mu^{F2}\left(\Pi_{s}\right)}{\partial w^{2}} & \frac{\partial^{2}\mu^{F2}\left(\Pi_{s}\right)}{\partial w\partial i_{s}}\\ \frac{\partial^{2}\mu^{F2}\left(\Pi_{s}\right)}{\partial i_{s}\partial w} & \frac{\partial^{2}\mu^{F2}\left(\Pi_{s}\right)}{\partial i_{s}^{2}} \end{array}\right)=\left(\begin{array}{cc} \frac{4B\left(1+2\lambda \right)\left(BH_{m}-k_{m}^{2}\right)}{\left(1+\lambda \right)\Delta_{m}} & \frac{2BH_{m}k_{s}}{\Delta_{m}}\\ \frac{2BH_{m}k_{s}}{\Delta_{m}} & -2H_{s} \end{array}\right)$$

Then the matrix determinant is

$$\begin{array}{c} \;\frac{4B\left(1+2\lambda \right)\left(BH_{m}-k_{m}^{2}\right)}{\left(1+\lambda \right)\Delta_{m}}\left(-2H_{s}\right)-{\left(\frac{2BH_{m}k_{s}}{\Delta_{m}}\right)}^{2}\\ =\frac{-4B}{\left(1+\lambda \right)\Delta_{m}^{2}}\left[2\left(1+2\lambda \right)\left(BH_{m}-k_{m}^{2}\right)H_{s}\Delta_{m}+\left(1+\lambda \right)BH_{m}^{2}k_{s}^{2}\right]>0 \end{array}$$

Therefore, this Hessian matrix is negative definite, which verified the objective function of the supplier’s profit is a joint concave function of $w^{F2}$ and $i_{s}^{F2}$. From the supplier’s matrix determinant we know $2\left(1+2\lambda \right)\left(BH_{m}-k_{m}^{2}\right)H_{s}\Delta_{m}+\left(1+\lambda \right)BH_{m}^{2}k_{s}^{2}>0$ holds the assumption. We insert the optimal $w^{F2*}$ and $i_{s}^{F2*}$ back into $p^{F2}\left(w,i_{s}\right)$ and $i_{m}^{F2}\left(w,i_{s}\right)$, then we get the optimal selling price of the manufacturer $p^{F2*}$,

$$\begin{array}{c} p^{F2*}=\frac{\left\{\begin{array}{c} \left(1-\lambda \right)\left(1+2\lambda \right)\left(2BH_{m}-k_{m}^{2}\right)\left(BH_{m}-k_{m}^{2}\right)H_{s}\Delta_{m}c+{\left(1+\lambda \right)}^{2}\left(2BH_{m}-k_{m}^{2}\right)H_{m}^{2}k_{s}^{2}c\\ +5\left(1+\lambda \right)\left(1+2\lambda \right)\left(2BH_{m}-k_{m}^{2}\right)BcH_{m}^{2}k_{s}^{2}-4\left(1+\lambda \right)\left(1+2\lambda \right)ABH_{m}^{2}H_{s}\Delta_{m}\\ +4{\left(1+\lambda \right)}^{2}ABH_{m}^{3}k_{s}^{2}-2\left(1+\lambda \right)\left(1+3\lambda \right)\left(BH_{m}-k_{m}^{2}\right)BcH_{m}^{2}k_{s}^{2} \end{array}\right\}}{\left(1+\lambda \right)\Delta_{m}\left[2\left(1+2\lambda \right)\left(BH_{m}-k_{m}^{2}\right)H_{s}\Delta_{m}+\left(1+\lambda \right)BH_{m}^{2}k_{s}^{2}\right]} \end{array}$$

the optimal sustainable green technology innovation effort of the manufacturer $i_{m}^{F2*}$.

$$\begin{array}{c} i_{m}^{F2*}=k_{m}\frac{\left\{\begin{array}{c} 2\left(1+\lambda \right)\left(1+2\lambda \right)\left(BH_{m}-k_{m}^{2}\right)AH_{s}\Delta_{m}+2{\left(1+\lambda \right)}^{2}ABH_{m}^{2}k_{s}^{2}\\ -\left(1+\lambda \right)\left(1+3\lambda \right)\left(BH_{m}-k_{m}^{2}\right)BcH_{m}k_{s}^{2}-\left(1-\lambda \right)\left(1+2\lambda \right)\left(BH_{m}-k_{m}^{2}\right)BcH_{s}\Delta_{m}\\ +{\left(1+\lambda \right)}^{2}B^{2}cH_{m}^{2}k_{s}^{2}-\left(1+\lambda \right)\left(1+2\lambda \right)ABH_{m}H_{s}\Delta_{m} \end{array}\right\}}{\left(1+\lambda \right)\Delta_{m}\left[2\left(1+2\lambda \right)\left(BH_{m}-k_{m}^{2}\right)H_{s}\Delta_{m}+\left(1+\lambda \right)BH_{m}^{2}k_{s}^{2}\right]} \end{array}$$

 ☐

**Proof** **of** **Proposition** **3.** If there is only the manufacturer concerning about fairness, The manufacturer’s sustainable green technology innovation effort is

$$\begin{array}{ccc} \frac{\partial i_{m}^{F2*}}{\partial \lambda } & = & \frac{\left\{\begin{array}{c} -\left(A-Bc\right)H_{m}k_{s}\left[\left(2-3\theta \right)H_{s}\Delta_{m}-\left(1-\theta \right)H_{m}k_{s}^{2}\right]\\ -\left(-3H_{s}\Delta_{m}+H_{m}k_{s}^{2}\right)\left[\left(1-\theta \right)\left(A-Bc\right)H_{m}k_{s}\right] \end{array}\right\}}{{\left[\left(2-3\theta \right)H_{s}\Delta_{m}-\left(1-\theta \right)H_{m}k_{s}^{2}\right]}^{2}}\\ & = & \frac{\left(A-Bc\right)H_{s}H_{m}k_{s}\Delta_{m}}{{\left[\left(2-3\theta \right)H_{s}\Delta_{m}-\left(1-\theta \right)H_{m}k_{s}^{2}\right]}^{2}}>0 \end{array}$$

The supplier’s sustainable green technology innovation effort is

$$\begin{array}{ccc} \frac{\partial i_{s}^{F2*}}{\partial \lambda } & = & \frac{\left\{\begin{array}{c} -\left(A-Bc\right)H_{m}k_{s}\left[\left(2-3\theta \right)H_{s}\Delta_{m}-\left(1-\theta \right)H_{m}k_{s}^{2}\right]\\ -\left(-3H_{s}\Delta_{m}+H_{m}k_{s}^{2}\right)\left[\left(1-\theta \right)\left(A-Bc\right)H_{m}k_{s}\right] \end{array}\right\}}{{\left[\left(2-3\theta \right)H_{s}\Delta_{m}-\left(1-\theta \right)H_{m}k_{s}^{2}\right]}^{2}}\\ & = & \frac{\left(A-Bc\right)H_{s}H_{m}k_{s}\Delta_{m}}{{\left[\left(2-3\theta \right)H_{s}\Delta_{m}-\left(1-\theta \right)H_{m}k_{s}^{2}\right]}^{2}}>0 \end{array}$$

 ☐
